# Identification of a Five-miRNA Signature for Diagnosis of Kidney Renal Clear Cell Carcinoma

**DOI:** 10.3389/fgene.2022.857411

**Published:** 2022-04-20

**Authors:** Enyang Zhao, Xuedong Li, Bosen You, Jinpeng Wang, Wenbin Hou, Qiong Wu

**Affiliations:** ^1^ School of Life Science and Technology, Harbin Institute of Technology, Harbin, China; ^2^ The Second Affiliated Hospital of Harbin Medical University, Harbin Medical University, Harbin, China; ^3^ State Key Laboratory of Urban Water Resource and Environment, Harbin Institute of Technology, Harbin, China

**Keywords:** kidney renal clear cell carcinoma, diagnosis, miRNA, signature, feature selection

## Abstract

**Motivation:** Kidney renal clear cell carcinoma, which is a common type and accounts for 70–80% of renal cell carcinoma, can easily lead to metastasis and even death. A reliable signature for diagnosis of this cancer is in need. Hence, we seek to select miRNAs for identifying kidney renal clear cell carcinoma.

**Method:** A feature selection strategy is used and improved to identify microRNAs for diagnosis of kidney renal clear cell carcinoma. Samples representing kidney renal clear cell carcinoma and normal tissues are split into training and testing groups. Accumulated scores representing the variable importance of each miRNA are derived from an iteration of resampling, training, and scoring. Those miRNAs with higher scores are selected based on the Gaussian mixture model. The sample split is repeated ten times to get more central miRNAs.

**Results:** A total of 611 samples are downloaded from TCGA, each of which contains 1,343 miRNAs. The improved feature selection method is implemented, and five miRNAs are identified as a biomarker for diagnosis of kidney renal clear cell carcinoma. GSE151419 and GSE151423 are selected as the independent testing sets. Experimental results indicate the effectiveness of the selected signature. Both data-driven measurements and knowledge-driven evidence are given to show the effectiveness of our selection results.

## 1 Introduction

Kidney renal clear cell carcinoma (KIRC), which can easily lead to metastasis and even death, is regarded as one of the most common cancers in adults (McDougal et al., 2006). In order to realize the molecular diagnosis or prognosis of KIRC, many discoveries about biomarkers or signatures of KIRC have been made (Zhan et al., 2015). Moreover, many corresponding data-mining tools have been made (Xie et al., 2019). MicroRNAs (miRNAs) are regarded as noncoding regulatory RNAs that regulate gene expressions by complementary binding with the 3′-untranslated region (UTR) of target mRNAs and causing their degradation or suppressing mRNA translation. The corresponding profiles have been used for discovering biomarkers associated with diagnosis of KIRC (White et al., 2011).

Focusing on diagnosis of KIRC, univariate differentially expressed analysis together with fold change is commonly used and still in use for finding differentially expressed genes between KIRC tumor and the adjacent normal samples (Yang et al., 2014; Cui et al., 2020). However, this univariate statistical testing is based on a univariate hypothesis, which has ignored the correlations among genes. As a result, it makes the subsequent consideration of diagnosis unreasonable using univariate differentially expressed genes together. Instead, multivariate statistics (e.g., multivariate hypothesis testings) or predictive models (e.g., multi-dimensional classifiers) are to be considered.

**FIGURE 1:**
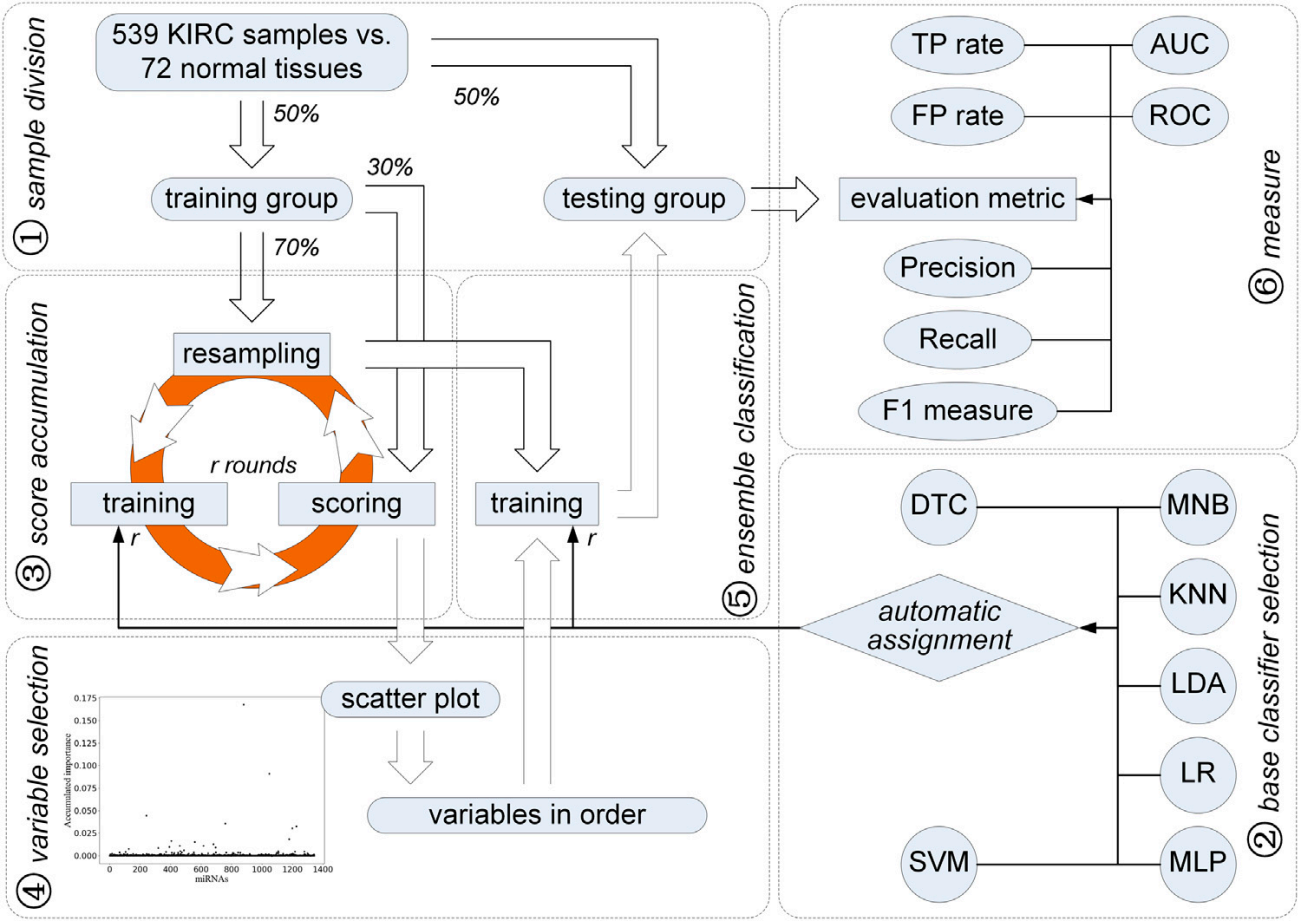
Improved feature selection strategy for identification of miRNAs for diagnosis of KIRC.

Different from univariate differentially expressed analysis, we applied and improved a feature selection strategy using ensemble classification, i.e., ECFS-DEA (Zhao et al., 2020), for finding miRNAs that play an important part in the diagnosis of KIRC, as illustrated in Figure 1. In addition to Fisher’s linear discriminant analysis (LDA), k-nearest-neighbor (KNN), support vector machine (SVM), and random forest, which is also named as decision tree classifier (DTC), logistic regression (LR) together with multi-layer perceptron (MLP), and multinomial naive Bayes (MNB) are also used. The samples are randomly split in balance within the KIRC and normal tissue groups. Using the improved feature selection strategy, 15 miRNAs are selected to be the candidates for the diagnosis of KIRC. In addition, five of them with higher rankings derived from the variable selection step in Figure 1 are chosen as a further signature. Both data-driven qualitative and quantitative measurements on the testing set and knowledge-driven KEGG pathway prediction demonstrate the effectiveness of the selected five miRNAs on diagnosis of KIRC.

## 2 Related Work

The most related work for finding a miRNA biomarker for the diagnosis of KIRC reported a three-microRNA signature (Liang et al., 2017). In fact, the panel of the three-miRNA was constructed following three steps. First of all, the unpaired *t*-test was considered to identify miRNAs that were differentially expressed between KIRC tumor and matched normal samples. Moreover, fold changes (FCs) in the expression of individual miRNA were calculated. The miRNAs with p < 0.05 and log_2_|*FCs*| > 2.0 were primarily screened out and considered to be significant. Therefore, a total of 63 differentially expressed miRNAs were individually identified. Second, the receiver operating characteristic (ROC) and the area under ROC curve (AUC) were calculated on each obtained miRNA. Those with better AUC values, i.e., ranging from 0.9–1.0, were regarded to have a better diagnostic performance. As a result, nine upregulated and 10 downregulated miRNAs were further selected. Third, Kaplan–Meier analysis with a log-rank test was used to evaluate the association between the miRNA expressions and patients’ survival time, that is, the samples were separated into two groups according to the expression values of each miRNA. Then, qualitative and quantitative results were obtained according to the Kaplan–Meier curves of the two groups and the corresponding log-rank test result. In this way, three miRNAs were ultimately selected as a signature.

As stated previously, all analyses, such as unpaired *t*-test, fold changes, AUC values, Kaplan–Meier analysis, and log-rank test, correspond to individual miRNAs. Based on the univariate hypothesis, these univariate statistical analyses make the discovered signature which is composed of several miRNAs and regarded as a multivariate variable become a logical contradiction. In contrast, we propose a method using the feature selection strategy using ensemble classification. Its technology roadmap is shown in Figure 1.

## 3 Methods

First of all, 611 transcriptome profiles including 539 cases of KIRC and 72 normal ones are downloaded from TCGA (gene expression quantification). Each sample contains 1,343 miRNA expression values after filtering 81 miRNAs with zero variance. Among them, those miRNAs which are involved in the discrimination between KIRC and normal samples need to be discussed further. In order to better express the improved feature selection method, we follow the steps shown in Figure 1.

### 3.1 Sample Division

In order to validate the effectiveness of the identified miRNAs, we make a balanced sample division. Samples within the KIRC and normal groups are equally split, that is, 50% of the samples are randomly selected as a training sample set, which contains not only KIRC samples but also normal tissues. The remaining half is regarded as the testing sample set. Moreover, the number of KIRC and normal samples in the training set is comparable to that in the testing set.

### 3.2 Resampling, Training, and Scoring

As shown in Figure 1, an iteration is implemented on the training group for obtaining miRNAs with higher accumulated scores. Each round of the iteration includes three steps, i.e., resampling, training, and scoring. First, 70% of the training samples are randomly chosen in a balanced way, that is, 70% of the KIRC samples and 70% of the normal ones are selected randomly for the subsequent training step. At the training step, these selected samples are used to train seven classifiers, i.e., DTC, MNB, KNN, LDA, LR, MLP, and SVM. All the miRNAs are considered. To evaluate the classification error rate, 30% of the left training samples are used, which is expressed as:

$$Err=\frac{\frac{FN}{TP+FN}+\frac{FP}{TN+FP}}{2},$$ (1)

where *FN*, *TP*, *FP,* and *TN* represent the number of false-negative, true-positive, false-positive, and true-negative samples, respectively. Here, KIRC tissues correspond to positive samples, and normal tissues correspond to negative ones. The classifier with the lowest classification error rate is assigned to be the chosen classifier in this round of resampling.

Supposing that no differential expression values occur between positive and negative samples on miRNA *i*, we make a permutation on its expression values. That is, the expression values of miRNA *i* are randomly reordered regardless of whether they belong to either positive samples or negative ones. Following Eq. 1, the classification error rate of the assigned classifier expressed as $\tilde{Err}$ is calculated using all the miRNAs. Correspondingly, the score of miRNA *i* is calculated as,

$$score_{j}\left(i\right)=\tilde{Err}-Err,$$ (2)

where *j* refers to the number of the iteration round. After *N* rounds of iteration, the accumulated score of miRNA *i* is expressed as,

$$Acc\text{\_}score\left(i\right)=\frac{\sum_{j=1}^{N}score_{j}\left(i\right)}{N}.$$ (3)

### 3.3 Variable Selection

After *N* rounds of resampling, training, and scoring, a scatter plot is obtained to show the accumulated score of each miRNA (see Figure 1). A double Gaussian mixture model (GMM) based on expectation maximization (EM) (Bishop, 2006) is used on these accumulated scores. The common boundary of the two Gaussian distributions representing the probability density function of these accumulated scores is regarded as a threshold. Those miRNAs with the accumulated score values higher than the threshold are chosen as the candidates for diagnosis of KIRC. Moreover, the order of the selected miRNA candidates can be determined according to their accumulated scores.

### 3.4 Establishing Ensemble Classifiers

According to the accumulated scores of the selected miRNAs, ensemble classifiers with different dimensions can be built. Classifiers including LDA, KNN, SVM, DTC, LR, MLP, and MNB have been used. Following the sample way as the resampling, training, and scoring step, a base classifier is trained and kept for further classification with the selected miRNA incrementally added according to their accumulated scores in descending order. To train and obtain the best base classifier with the lowest classification error rate, 70% of the KIRC samples and the normal ones are randomly selected. Each round of the resampling and training steps helps to obtain a base classifier. Thus, the ensemble classifiers on different dimensions are built.

### 3.5 Measurements

In order to validate the effectiveness of the selected miRNAs, we make seven measurements on the testing set. Based on the confusion matrix, which is composed of *TP*, *FN*, *TN,* and *FP*, five quantitative measurements, i.e., *T P rate*, *F P rate*, *Precision*, *Recall,* and *F*1 − *measure* are computed as follows,

$$\begin{array}{c} T\;P\;rate=\frac{TP}{TP+FN};\\ F\;P\;rate=\frac{FP}{FP+TN};\\ Precision=\frac{TP}{TP+FP};\\ Recall=\frac{TP}{TP+FN};\\ F1-measure=\frac{2*Precision*Recall}{Precision+Recall} \end{array},$$ (4)

where *T P rate* and *Recall* are expressed in the same form. In addition, two more qualitative and quantitative measurements, i.e., the receiver operating characteristic (ROC) and the area under ROC curve (AUC), are utilized.

## 4 Results

Experiments were conducted on 611 transcriptome profiles including 539 cases of KIRC cells and 72 normal ones downloaded from TCGA (https://portal.gdc.cancer.gov/repository). In order to achieve stable and reliable results, we repeated the procedure shown in Figure 1 ten times, that is, the sample division was randomly made ten times, each of which corresponded to a new pair of training and testing sets. Moreover, GSE15149 with 58 cancer and 17 normal samples and GSE151423 with 26 cancer and six normal samples were downloaded (https://www.ncbi.nlm.nih.gov) and used as the independent testing sets.

### 4.1 Data-Driven Results on 15 Selected miRNA Candidates

**FIGURE 2:**
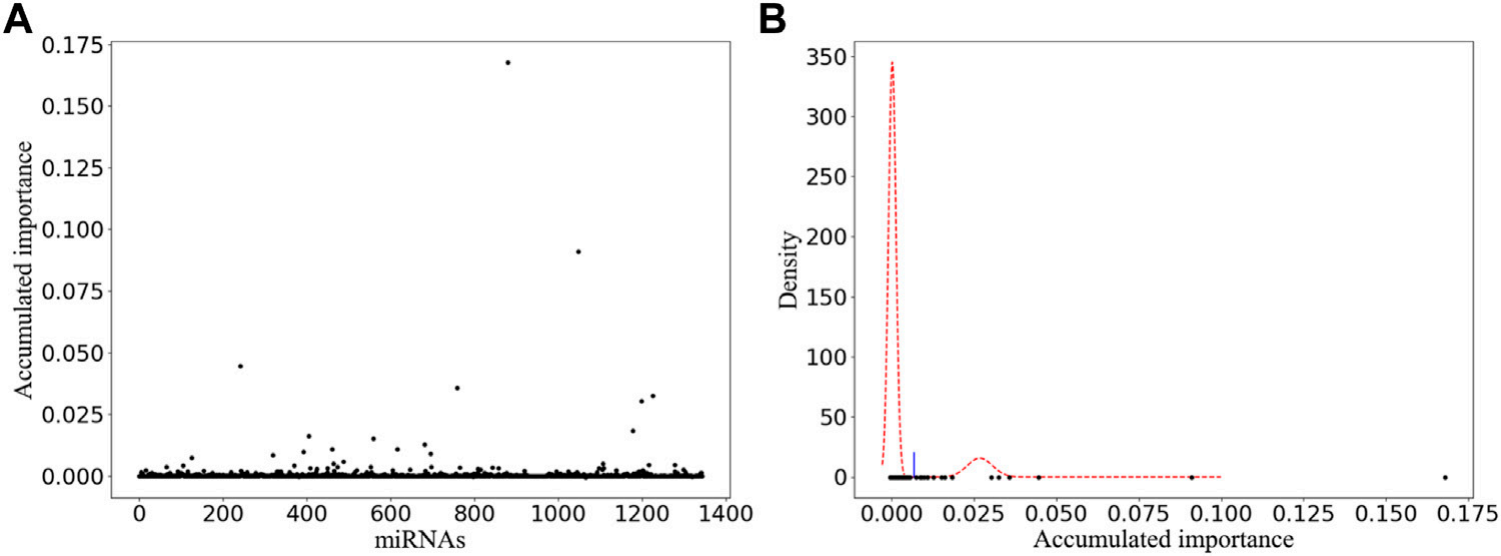
Variable selection result of one-time sample division. **(A)** Obtained scatter plot. Its horizontal and vertical ordinates correspond to the labels of miRNAs and their accumulated scores, respectively. **(B)** Corresponding miRNA selection result. Its horizontal and vertical ordinates refer to the accumulated scores and the corresponding probability density, respectively.

In order to stabilize the experimental results obtained by our improved miRNA selection method, we performed 1 × 10^5^ rounds of resampling, training, and scoring. The details of the obtained scatter plot and the corresponding miRNA selection result based on double GMM with EM algorithm are listed in Figure 2A and Figure 2B, respectively. A total of 15 miRNAs are selected, which are shown as the scatter points on the right side of the threshold (the blue line) in Figure 2B. Among them, we manually selected six miRNAs with their accumulated scores higher than 0.025.

Focusing on the miRNA with the highest accumulated score, the six miRNAs, 15 miRNAs, and all the miRNAs in existence, we alternatively established the ensemble classifiers after 1 × 10^4^ rounds of resampling and training steps and calculated the confusion matrix and the corresponding quantitative measurements expressed in Eq. 4 on the testing set. The experimental results are listed in Table 1. It can be found that several miRNAs may achieve comparable classification results as all miRNAs do, which indicates that miRNA biomarkers probably do exist for diagnosis of KIRC.

**TABLE 1:** Quantitative results after variable selection corresponding to one-time sample division.

Feature	Confusion matrix			Class	TP rate	FP rate	Precision	Recall	F1-measure
miR-621	Classified as − >	a	b	a: positive	0.686	0.052	0.632	0.686	0.658
	a	24	11	b: positive	0.948	0.314	0.959	0.948	0.954
	b	14	257	Weighted average	0.918	0.284	0.922	0.918	0.920
miR-{621,140,570,210,3189,1270}	Classified as − >	a	b	a: positive	0.943	0.044	0.733	0.943	0.825
	a	33	2	b: positive	0.956	0.057	0.992	0.956	0.974
	b	12	259	Weighted average	0.955	0.056	0.962	0.955	0.957
miR-{621,140,570,210,3189,1270	Classified as − >	a	b	a: positive	0.943	0.018	0.868	0.943	0.904
647,4645,126,25,4664,4457	a	33	2	b: positive	0.982	0.057	0.993	0.982	0.987
4477B,3682,3609}	b	5	266	Weighted average	0.978	0.053	0.979	0.978	0.978
All 1,343 miRNAs	Classified as − >	a	b	a: positive	0.943	0.030	0.805	0.943	0.868
	a	33	2	b: positive	0.970	0.057	0.992	0.970	0.981
	b	8	263	Weighted average	0.967	0.054	0.971	0.967	0.968

**FIGURE 3:**
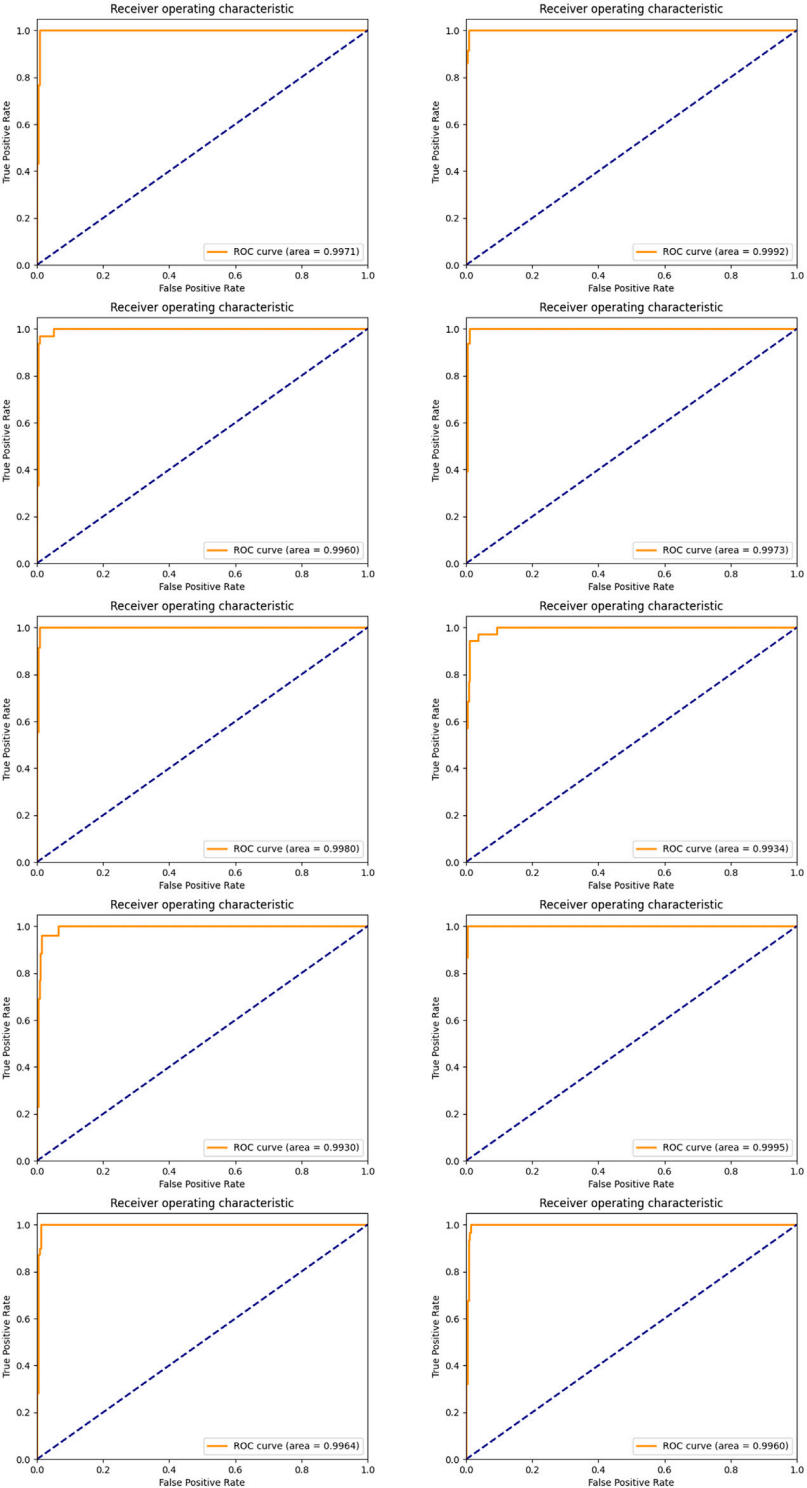
ROC curves and the corresponding AUCs of selected miRNA candidates after ten-time sample division.

**FIGURE 4:**
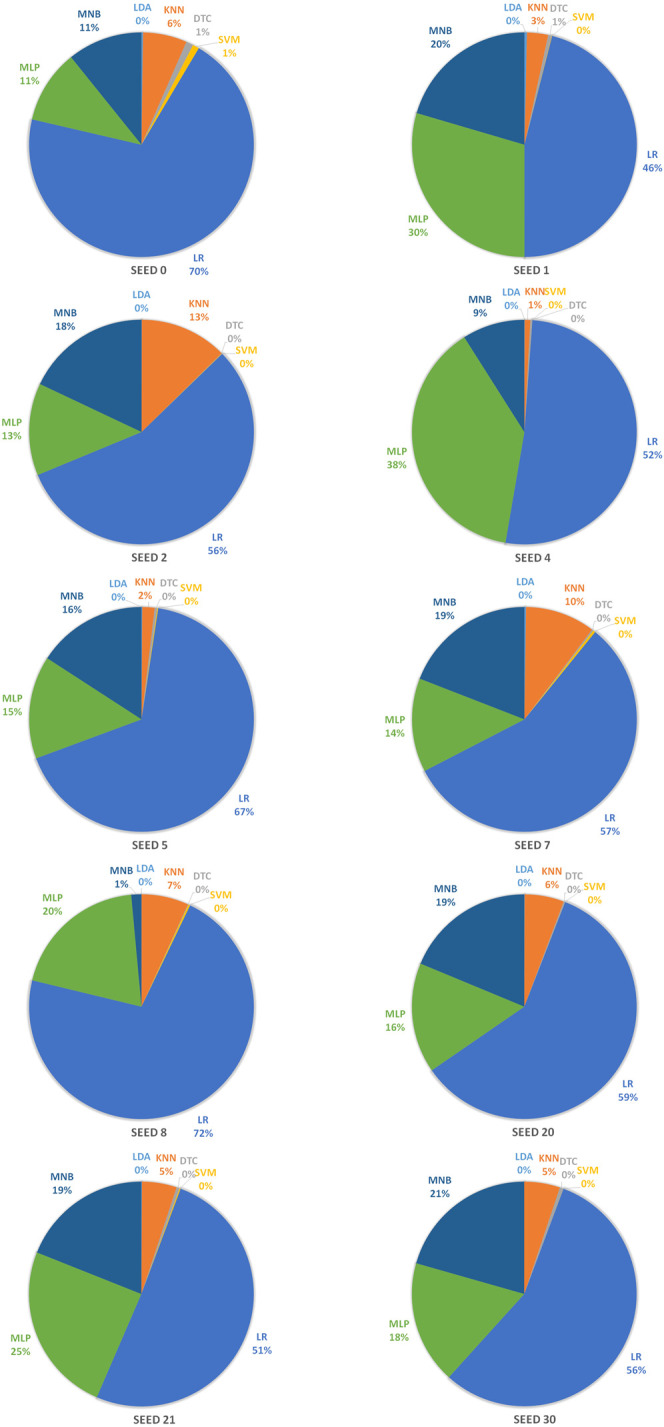
Pie charts representing the contribution of seven base classifiers according to ten times of the sample division.

In order to achieve stable miRNA biomarkers, sample division was randomly performed ten times. The corresponding ROCs and AUCs are listed in Figure 3. Each ROC and AUC show that the selected miRNAs keep a high qualified classification result. In addition, ten pie charts were made (see Figure 4) corresponding to ten times of the sample division, each of which indicated the contribution of the seven base classifiers to the score accumulation step. It can be seen in Figure 4 that LR, MLP, and MNB play an important role in turn, which indicates a different classifier is to be considered because of different sample distributions.

### 4.2 Data-Driven Results on a Five-miRNA Signature

**FIGURE 5:**
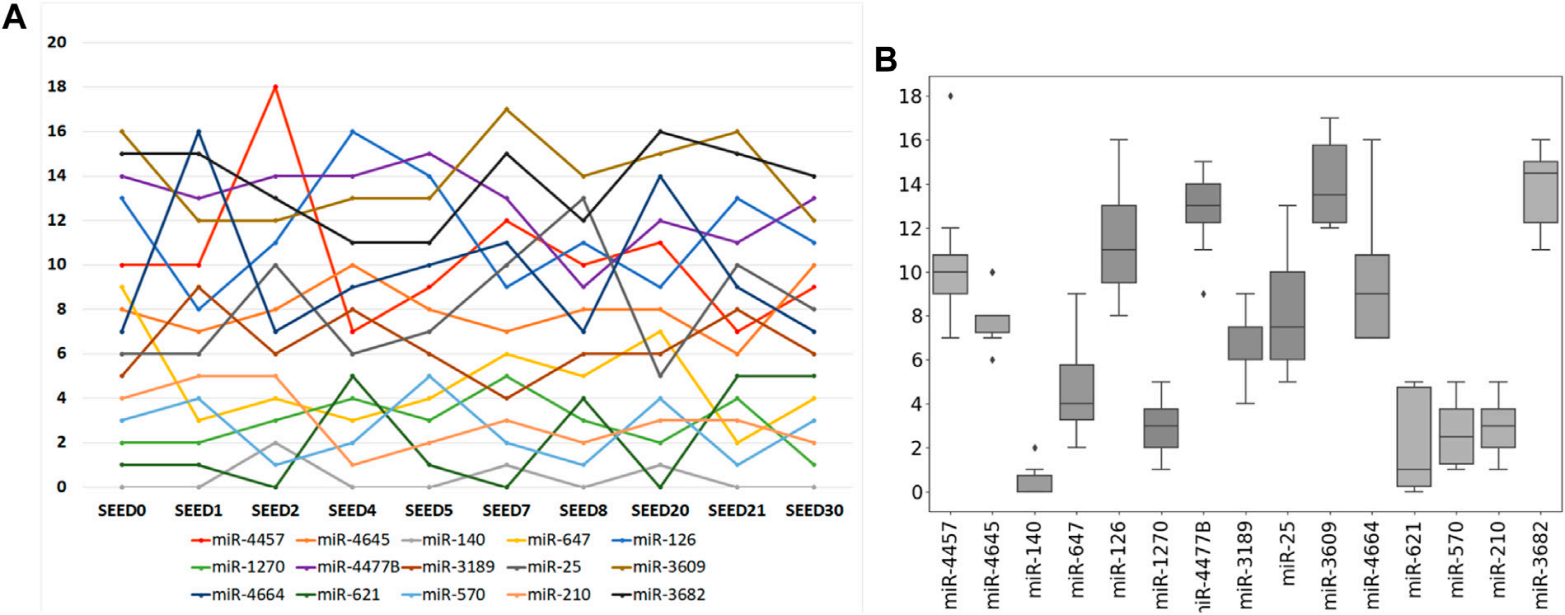
Ranking of each selected miRNA according to ten times of the sample division. **(A)** Line chart of each selected miRNA. Its horizontal and vertical ordinates refer to ten times of sample split and the accumulated score orders of the selected miRNAs, respectively. **(B)** Corresponding box diagram of each selected miRNA.

Furthermore, an intersection is made among the selected miRNAs derived from ten times of the sample division. The result is that the previous 15 miRNAs just constitute the intersection of the selected miRNAs from ten times of the sample division. As shown in Figure 5, the ranking of each miRNA in the miRNA intersection is listed according to the ten times of the sample division. Figure 5A refers to the line chart of each selected miRNA. The horizontal coordinate-axis *X* represents ten times of the sample division, whereas the vertical coordinate-axis *Y* refers to the ranking of the selected miRNA at each time of the sample division. Figure 5B shows the box diagram of each selected miRNA. The horizontal coordinate-axis *X* refers to each selected miRNA, whereas, the vertical coordinate-axis *Y* also represents the ranking of the selected miRNA at each time of the sample division. A total of five miRNAs, i.e., miR-140, miR-1270, miR-621, miR-570, and miR-210, can be selected as a further signature, for their rankings are within the first five.

**FIGURE 6:**
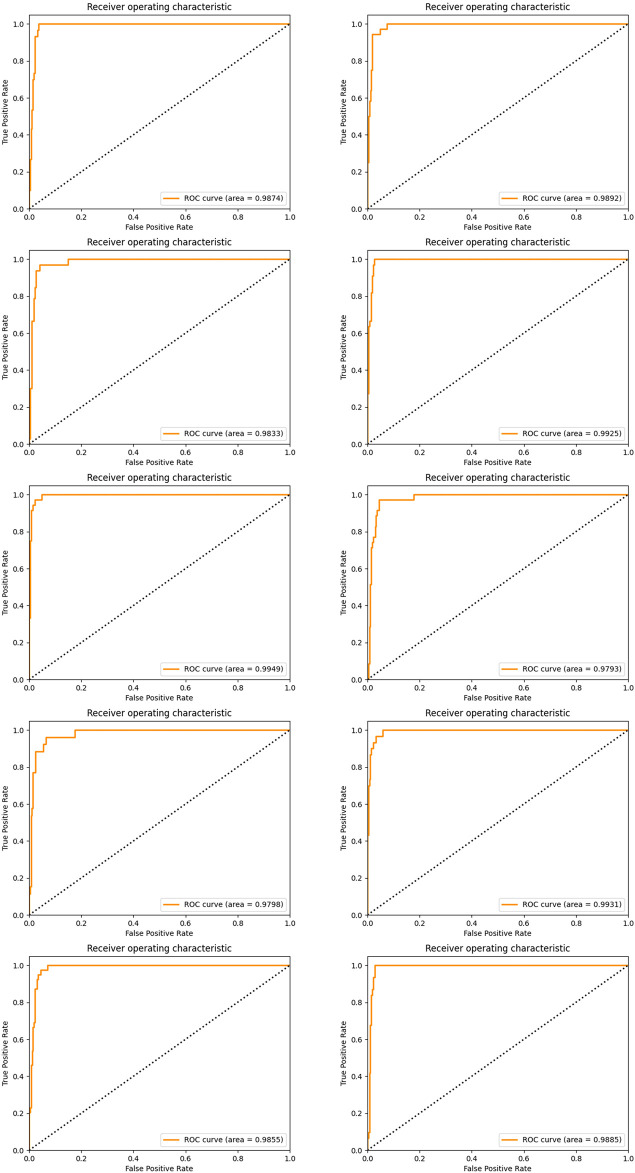
ROC curves and the corresponding AUCs of a five-miRNA signature after ten-time sample division.

Again, we established the ensemble classifier in the dimensional space derived from the selected five miRNAs after 1 × 10^4^ rounds of resampling and training steps and got the ROC curves and corresponding AUCs on each testing set derived from ten times of the sample division. The results are illustrated in Figure 6. By making a careful comparison between the results in Figure 3 and Figure 6, we conclude that miR-140, miR-1270, miR-621, miR-570, and miR-210 may form a five-miRNA signature for diagnosis of KIRC.

In addition, we made a comparison between the multivariate five-miRNA signature we obtained and the previously obtained univariate three-miRNA signature (Liang et al., 2017). In the univariate three-miRNA signature, miR-21 and miR-155 were not shown in gene expression quantification data. As for miR-584, it was well expressed. On account of its univariate discriminative property, the ensemble classifiers were alternatively built after rounds of resampling and training steps. The corresponding classification results on miR-584 and our selected five miRNAs are shown in Table 2. The experimental result shows that the obtained five miRNAs constitute a better signature for diagnosis of KIRC.

**TABLE 2:** Quantitative results between the obtained five-miRNA signature and the previously discovered miRNA (Liang et al., 2017).

Feature	Confusion matrix			Class	TP rate	FP rate	Precision	Recall	F1-measure
miR-584	Classified as − >	a	b	a: positive	1.000	0.944	0.124	1.000	0.220
	a	36	0	b: positive	0.056	0.000	1.000	0.056	0.105
	b	235	15	Weighted average	0.164	0.108	0.900	0.164	0.118
miR-{621,140,570,210,1270}	Classified as − >	a	b	a: positive	1.000	0.081	0.621	1.000	0.766
	a	36	0	b: positive	0.919	0.000	1.000	0.919	0.958
	b	22	248	Weighted average	0.928	0.009	0.957	0.928	0.936

### 4.3 Knowledge-Driven Results on the Five-miRNA Signature

Next, we dedicate to finding knowledge-driven evidence that the five-miRNA signature works in diagnosis of KIRC. DIANA-miRPath v3.0 (Vlachos et al., 2015) was used as the tool for finding associated pathways and target genes. Using Tarbase, TargetScan, and microT-CDS as the target prediction tool with default parameters, we found that the pathway “renal cell carcinoma” stands in the second place after searching “hsa-miR-140-5p” with the gene intersection set to three by default. Furthermore, we searched all the five miRNAs again using Tarbase as the target prediction tool with default parameters and found that pathway again.

**FIGURE 7:**
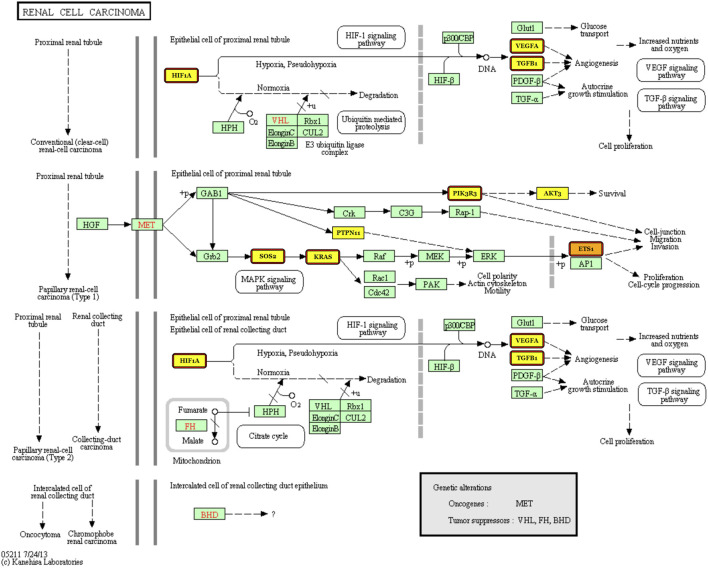
Renal cell carcinoma pathway with the targets regulated by miR-140, miR-1270, and miR-570 labeled.

Figure 7 illustrates that the targets are regulated by miR-140, miR-1270, and miR-570, which means that the three miRNAs from the five-miRNA signature have been reported to be associated with KIRC. Targets with highlights are those regulated by miR-140, miR-1270, and miR-570, while those with red borders are the targets regulated by miR-140. It can be indicated that miR-140, which ranks first (see Figure 5B), participates in four kinds of carcinogenesis in renal cell carcinoma. This corresponds to the use of all 611 cases composed of 539 KIRC cases and 72 normal ones without considering any cancer stage in advance.

**FIGURE 8:**
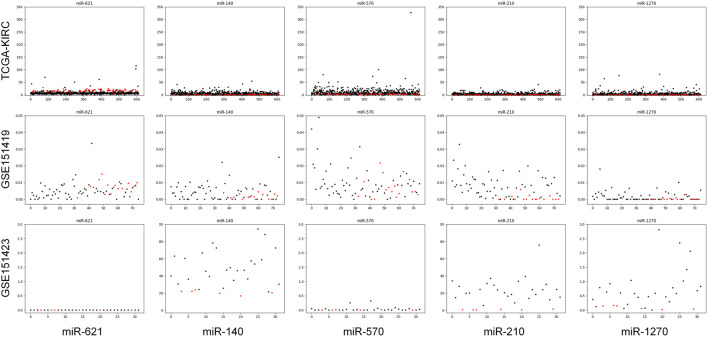
Scatter plots of the expression levels corresponding to each of the five miRNAs on TCGA, GSE151419, and GSE151423.

**FIGURE 9:**
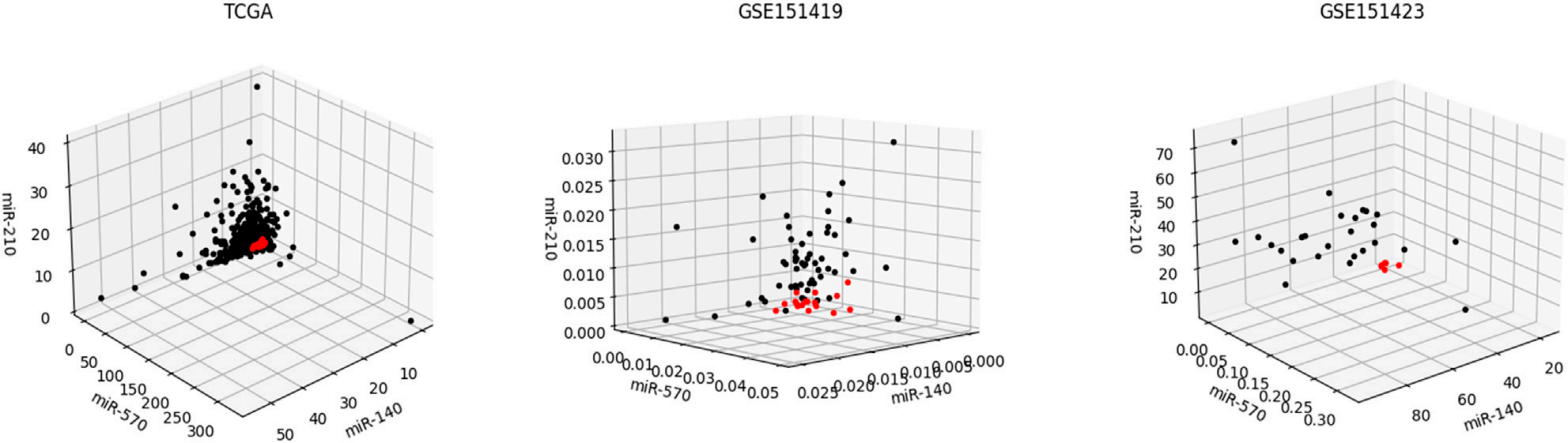
Scatter plots of the expression levels corresponding miR-140, miR-570, and miR-210 on TCGA, GSE151419, and GSE151423.

Moreover, it needs to be discussed whether the five-miRNA signature still works on cross-platform data. We chose GSE151419 and GSE151423 and found that the established ensemble classifier using TCGA data lapsed. Because of the poor classification results, we made scatter plots of the expression levels corresponding to each of the five miRNAs on TCGA, GSE151419, and GSE151423. As shown in Figure 8, it can be found that the expression levels from the three data are not on the same scale. That means it is the systematical error deriving from different sample sets that makes poor classification results. In addition, we tried all combinations of the five miRNAs and found that miR-140, miR-570, and miR-210 keep separable scatter plots between cancer samples and normal ones, as shown in Figure 9. The corresponding quantitative results are shown in Table 3.

**TABLE 3:** Quantitative results on TCGA and GSE151423 using miR-140, miR-570, and miR-210.

Training data	Testing data	Confusion matrix			Class	TP rate	FP rate	Precision	Recall	F1-measure
TCGA	TCGA	Classified as − >	a	b	a: positive	1.000	0.107	0.554	1.000	0.713
		a	36	0	b: positive	0.893	0.000	1.000	0.893	0.943
		b	29	241	Weighted average	0.906	0.012	0.947	0.906	0.916
GSE151419	GSE151423	Classified as − >	a	b	a: positive	1.000	0.038	0.857	1.000	0.923
		a	6	0	b: positive	0.962	0.000	1.000	0.962	0.980
		b	1	25	Weighted average	0.940	0.007	0.973	0.969	0.969

## 5 Discussion

We used gene expression quantification from TCGA other than miRNA expression quantification, due to the sparse form of the expression levels derived from the latter one. According to the experimental results, we plan to make three discussions as follows. First, it needs to be discussed whether ten times of the sample division is enough. From Figure 5B, we can see that miR-140 stays firm near the top. However, miR-621 appears in the first place three times out of the ten times of sample division (see Figure 5A). Instead of a random sample division, we also made 10-fold cross-validation and found that miR-140 stays firm in the first place. That probably means we need more samples (especially more normal cases) for training. Therefore, when the sample size is small, it would be wiser to make n-fold cross-validation.

Second, whether the five miRNAs regarded as the signature have been reported needs to be discussed. To the best of our knowledge, we have found that miR-140 (Zhang and Lu, 2020) and miR-210 (Nakada et al., 2020) are reported to be associated with KIRC. As for the regulated targets in the renal cell carcinoma pathway, TGFB1 (Song et al., 2018), EPAS1 (Cho et al., 2016), HIF1A (Shen et al., 2011), VEGFA (Sun et al., 2020), KRAS, and PIK3CA (Lee et al., 2020) regulated by miR-140 have been reported to interfere with KIRC. As for miR-1270, there is also a report about AKT3 (Fan et al., 2020) that is associated with KIRC. This means that the five selected miRNAs are a new and useful signature to distinguish KIRC cases from the normal ones.

Third, the experimental results are only statistically proven because of the lack of *in vitro* and *in vivo* biological verification. Anyway, the pathway “renal cell carcinoma” is selected (please see Figure 7) according to the identified statistical signature, which gives our data-driven result a knowledge-driven support. A biological verification including not only *in vitro* and *in vivo* experiments but also plasma sampling is to be performed in our future work.

## 6 Conclusion

MicroRNAs may play a vital role in diagnosis of kidney renal clear cell carcinoma. In this study, we used and improved a feature selection method to select microRNAs for the diagnosis of this disease. Samples representing kidney renal clear cell carcinoma and normal cases were split into training and testing sets. An iteration referred to resampling, training, and scoring steps was implemented to stabilize the results of feature selection. MicroRNAs with higher rankings were selected according to the Gaussian mixture model based on expectation maximization. Qualitative and quantitative results demonstrated that miR-140 plays an important role in predicting kidney renal clear cell carcinoma. In addition, a five-miRNA signature is obtained for diagnosis of kidney renal clear cell carcinoma.

## Data Availability

Publicly available datasets were analyzed in this study. These data can be found at: https://portal.gdc.cancer.gov/repository.

## References

[B1] Bishop C. (2006). Pattern Recognition and Machine Learning. Singapore: Springer.

[B2] Cho H. Du X. Rizzi J. P. Liberzon E. Chakraborty A. A. Gao W. (2016). On-target Efficacy of a HIF-2α Antagonist in Preclinical Kidney Cancer Models. Nature 539, 107–111. 10.1038/nature19795 27595393 PMC5499381

[B3] Cui H. Shan H. Miao M. Z. Jiang Z. Meng Y. Chen R. (2020). Identification of the Key Genes and Pathways Involved in the Tumorigenesis and Prognosis of Kidney Renal clear Cell Carcinoma. Sci. Rep. 10, 4271. 10.1038/s41598-020-61162-4 32144299 PMC7060270

[B4] Fan D. Liu Q. Wu F. Liu N. Qu H. Yuan Y. (2020). Prognostic Significance of PI3K/AKT/mTOR Signaling Pathway Members in clear Cell Renal Cell Carcinoma. PEERJ 8, e9261. 10.7717/peerj.9261 32547875 PMC7271881

[B5] Lee H. J. Shin D. H. Park J. Y. Kim S. Y. Hwang C. S. Lee J. H. (2020). Unilateral Synchronous Papillary Renal Neoplasm with Reverse Polarity and clear Cell Renal Cell Carcinoma: a Case Report with Kras and Pik3ca Mutations. Diagn. Pathol. 15, 123. 10.1186/s13000-020-01042-7 33023600 PMC7539524

[B6] Liang B. Zhao J. Wang X. (2017). A Three-Microrna Signature as a Diagnostic and Prognostic Marker in clear Cell Renal Cancer: an In Silico Analysis. PLoS One 12, e0180660. 10.1371/journal.pone.0180660 28662155 PMC5491330

[B7] McDougal W. S. Tolkoff-Rubin N. E. Michaelson M. D. Mueller P. R. Braaten K. (2006). Case 28-2006. N. Engl. J. Med. 355, 1161–1167. 10.1056/NEJMcpc069019 16971723

[B8] Nakada C. Hijiya N. Tsukamoto Y. Yano S. Kai T. Uchida T. (2020). A Transgenic Mouse Expressing miR‐210 in Proximal Tubule Cells Shows Mitochondrial Alteration: Possible Association of miR‐210 with a Shift in Energy Metabolism. J. Pathol. 251, 12–25. 10.1002/path.5394 32073141

[B9] Shen C. Beroukhim R. Schumacher S. E. Zhou J. Chang M. Signoretti S. (2011). Genetic and Functional Studies Implicate HIF1α as a 14q Kidney Cancer Suppressor Gene. Cancer Discov. 1, 222–235. 10.1158/2159-8290.CD-11-0098 22037472 PMC3202343

[B10] Song E. Song W. Ren M. Xing L. Ni W. Li Y. (2018). Identification of Potential Crucial Genes Associated with Carcinogenesis of clear Cell Renal Cell Carcinoma. J. Cel. Biochem. 119, 5163–5174. 10.1002/jcb.26543 29227586

[B11] Sun J. Tang Q. Gao Y. Zhang W. Zhao Z. Yang F. (2020). VHL Mutation-Mediated SALL4 Overexpression Promotes Tumorigenesis and Vascularization of clear Cell Renal Cell Carcinoma via Akt/GSK-3β Signaling. J. Exp. Clin. Cancer Res. 39, 104. 10.1186/s13046-020-01609-8 32513235 PMC7278163

[B12] Vlachos I. S. Zagganas K. Paraskevopoulou M. D. Georgakilas G. Karagkouni D. Vergoulis T. (2015). Diana-mirpath v3.0: Deciphering Microrna Function with Experimental Support. Nucleic Acids Res. 43, W460–W466. 10.1093/nar/gkv403 25977294 PMC4489228

[B13] White N. M. A. Bao T. T. Grigull J. Youssef Y. M. Girgis A. Diamandis M. (2011). Mirna Profiling for clear Cell Renal Cell Carcinoma: Biomarker Discovery and Identification of Potential Controls and Consequences of Mirna Dysregulation. J. Urol. 186, 1077–1083. 10.1016/j.juro.2011.04.110 21784468

[B14] Xie L. Wang Q. Dang Y. Ge L. Sun X. Li N. (2019). Oskirc: a Web Tool for Identifying Prognostic Biomarkers in Kidney Renal clear Cell Carcinoma. Future Oncol. 15, 3103–3110. 10.2217/fon-2019-0296 31368353

[B15] Yang W. Yoshigoe K. Qin X. Liu J. S. Yang J. Y. Niemierko A. (2014). Identification of Genes and Pathways Involved in Kidney Renal clear Cell Carcinoma. BMC Bioinformatics 15, S2. 10.1186/1471-2105-15-S17-S2 PMC4304191 25559354

[B16] Zhan Y. Guo W. Zhang Y. Wang Q. Xu X.-j. Zhu L. (2015). A Five-Gene Signature Predicts Prognosis in Patients with Kidney Renal clear Cell Carcinoma. Comput. Math. Methods Med. 2015, 1–7. 10.1155/2015/842784 PMC4619904 26539246

[B17] Zhang Y. J. Lu C. (2020). Long Non-coding Rna Hcp5 Promotes Proliferation and Metastasis of clear Cell Renal Cell Carcinoma via Targeting Mir-140-5p/igf1r Pathway. Eur. Rev. Med. Pharmacol. Sci. 24, 2965–2975. 10.26355/eurrev_202003_20661 32271414

[B18] Zhao X. Jiao Q. Li H. Wu Y. Wang H. Huang S. (2020). Ecfs-dea: an Ensemble Classifier-Based Feature Selection for Differential Expression Analysis on Expression Profiles. BMC Bioinformatics 21, 43. 10.1186/s12859-020-3388-y 32024464 PMC7003361

